# Optimization of Bone Scaffold Porosity Distributions

**DOI:** 10.1038/s41598-019-44872-2

**Published:** 2019-06-24

**Authors:** Patrina S. P. Poh, Dvina Valainis, Kaushik Bhattacharya, Martijn van Griensven, Patrick Dondl

**Affiliations:** 1 Department of Experimental Trauma Surgery, Klinikum Rechts der Isar, Technische Universität München, Munich, Germany; 2 Julius Wolff Institute for Biomechanics and Musculoskeletal Regeneration, Charité – Univeristätsmedizin Berlin, Berlin, Germany; 3 Division of Engineering and Applied Sciences, California Institute of Technology, Pasadena, CA USA; 4 Abteilung für Angewandte Mathematik, Albert-Ludwigs-Universität Freiburg, Freiburg, Germany

**Keywords:** Applied mathematics, Preclinical research

## Abstract

Additive manufacturing (AM) is a rapidly emerging technology that has the potential to produce personalized scaffolds for tissue engineering applications with unprecedented control of structural and functional design. Particularly for bone defect regeneration, the complex coupling of biological mechanisms to the scaffolds’ properties has led to a predominantly trial-and-error approach. To mitigate this, shape or topology optimization can be a useful tool to design a scaffold architecture that matches the desired design targets, albeit at high computational cost. Here, we consider an efficient macroscopic optimization routine based on a simple one-dimensional time-dependent model for bone regeneration in the presence of a bioresorbable polymer scaffold. The result of the optimization procedure is a scaffold porosity distribution which maximizes the stiffness of the scaffold and regenerated bone system over the entire regeneration time, so that the propensity for mechanical failure is minimized.

## Introduction

The regeneration and restoration of skeletal functions of critical-sized bone defects are very challenging. Presently, regenerative therapy using absorbable scaffolds have shown promising results *in vivo*^1–5^ and in clinical cases^6–9^. During the bone regeneration processes, scaffolds act as a temporary supporting structure to *(a)* ensure that the defect/regeneration space is suitable for bone tissue growth, maturation and remodeling; *(b)* provide mechanical functionality for proper transfer of loads acting on scaffolds to the adjacent host tissues while tissue regenerates; and *(c)* facilitate in-growth of tissue and vasculature to accelerate tissue regeneration. Hence, the scaffolds’ architecture plays a crucial role during bone regeneration processes. Fundamentally, in the design of bone scaffolds, there are several functional characteristics to be considered, such as the porosity (or, inversely, the biomaterial volume fraction), pore size and pore shape, as these will affect the scaffolds’ permeability/diffusivity, degradation rate and elastic modulus, and in turn the biological processes necessary for regeneration^10^.

Due to the intricate relationships between scaffold geometries, mechanical properties, biomaterials and biological processes, research in bone tissue engineering has been dominated by trial-and-error approach – whereby an existing design is modified based on the experimental outcomes. This approach usually requires costly protocols and time-consuming experiments. Over the years, due to the rapid development of computer-aided engineering (CAE) tools, topology optimization techniques have shown their potential as a powerful tool for the design of scaffold architectures. Topology optimization is a numerical process that iteratively distributes a given amount of material within a given design space under specific constraints such that the final structure meets specific design targets^11,12^. This technique has been applied to design scaffolds to topologically achieve the optimized architecture to match the desired porosity, elastic modulus and fluid permeability^13–18^.

One limitation common to most CAE models for topology optimization of bone scaffolds architectures is that the time-dependence of the regeneration process is not taken into account. Bone regeneration using scaffolds is a complex phenomenon involving different biophysical processes and scaffold/tissue interactions via their elastic moduli over time. To this end, various multi-scale models have been proposed and developed simulating bone regeneration processes in response to various scaffold properties (e.g., porosity, permeability, elastic modulus), taking into account the time-evolution of the microstructure due to bone growth and scaffold resorption^19^. Hollister *et al*.^20^ proposed a topology optimization approach that can design a scaffold microstructure in order to meet the resulting conflicting design requirements.

However, implementation of CAE into routine additive manufacturing (AM) workflows for optimization of scaffolds for regenerative medicine is impeded by the high computational cost. Here, we propose a simple, one-dimensional model for the optimization of bone scaffold architectures based on homogenized quantities. The main advantage of this model will be its efficiency for numerical computation. We can, therefore, use it as a first test case for a scaffold shape-optimization procedure, in particular with respect to finding suitable objective functions to optimize. Our approach is thus related to the first step in the “Shape Optimization by the Homogenization Method”^11^ of optimizing within a relaxed problem for averaged quantities.

The remainder of this article is organized as follows. In the next section we introduce our simplified bone regeneration model and show some comparison to *in-vivo* studies. The section “Scaffold density optimization” describes the numerical optimization routine. The results of our numerical study and some discussion are presented thereafter. Finally, we present some conclusions and give an outlook on future work.

## Development of a Scaffold-Mediated Bone Regeneration Model

In bone tissue engineering, the process of bone regeneration is commonly mediated by osteoconductive scaffolds that are often combined with growth factors and/or cells (osteoinductive). However, the exact mechanisms by which scaffold-mediated bone regeneration occurs are yet unclear. The process of bone regeneration is a complex and continuous process, but well orchestrated, starting from the formation of hematoma accompanied by the infiltration of inflammatory cells. Following the events of inflammation, bone regeneration can occur through either endochondral (bone formation through an intermediate cartilage phase) or intramembranous ossification (formation of new bone directly – usually occuring adjacent to existing bone). Finally, the regenerating bone enters the remodeling stage, where newly formed bone continues to remodel itself until a mechanically strong and highly organized bone structure is restored. The specific mechanism of bone regeneration guided by scaffolds is determined by the scaffolds’ architecture, which provides the immediate mechanobiological and chemical environment where the cells reside.

For example, using collagen-based scaffolds (fabricated via freeze-drying techniques) with cylindrical pores aligned along the principal stress axis, Peterson *et al*.^21^ demonstrated the possibility of using solely the architecture of scaffolds as a physical guiding structure to promote endochondral healing of bone defects in rats. Cipitria *et al*. and Paris *et al*.^22,23^ illustrated that 3D-printed scaffolds made from a composite of polycaprolactone (PCL, a slowly degrading synthetic thermoplastic) and β-tricalcium phosphate (β-TCP) with rectangle-shaped pores guide new bone formation through intramembranous ossification in sheep, extending from the proximal and distal end of the osteotomy site parallel to the tibial axis. In these studies^21–23^, it was noted that in the absence of exogenous growth factors or cells, no clinically relevant bridging of the defect was achieved. However, Pobloth *et al*.^24^ illustrated that 3D-printed titanium scaffolds with optimized mechanobiological properties can promote clinically relevant functional bridging of a critical-sized bone defect in a large animal model without the addition of exogenous growth factors or cells. Collectively, these studies indicate the possibility that scaffolds with optimal architecture and mechanobiological properties can guide and support endogenous bone regeneration.

In this study, the primary intention of the proposed scaffold-mediated bone regeneration model is to provide a basis for the scaffold architecture optimization algorithm proposed in the section “Scaffold density optimization”. Hence, we propose a simple mathematical model capturing specific events occurring during the process of scaffold-mediated bone regeneration. Additionally, the model does take into consideration the time aspect of tissue regeneration, considering both bone growth and scaffold degradation and the interaction of the two. The time evolution model tracks the time dependent quantities relevant for the mechanical integrity of the scaffold. These quantities are the molecular weight of the scaffold material (which diminishes over time due to degradation) and the amount of bone regenerated. The bone regeneration, however, depends on the local microenvironment. This includes *(a)* the presence of endogenous angiogenic and osteoinductive factors (e.g., growth factors/cytokines), which are excreted into, diffuse through, and decay in the extracellular matrix in the interstitial space, creating a local gradient through the regenerating tissue; and *(b)* the mechanical strain stimulus transmitted through the structure due to external mechanical loading, as it is well established that mechanical stimuli play a major role in bone regeneration^25^.

### The system of equations for scaffold-mediated bone regeneration

**Figure 1:**
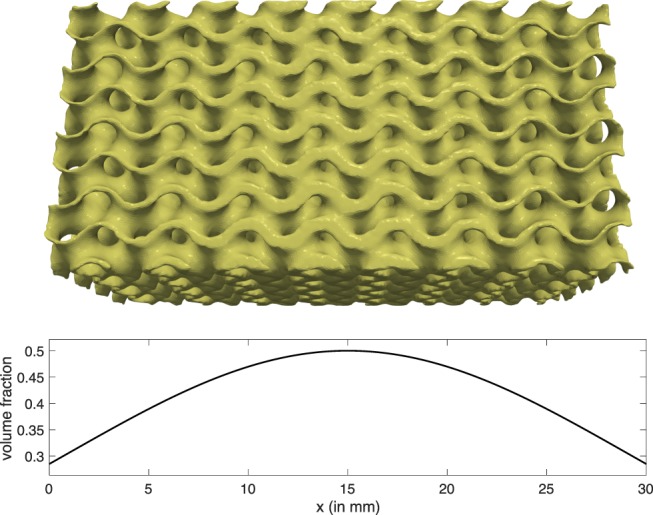
Coarse-Graining: above, a scaffold with varying local density; below, the coarse grained description of the local density.

A coupled system of evolution equations for these quantities, together with boundary conditions, has been established and constants in the model (e.g., the degradation rate of the scaffold material) have been deduced from experimental observations. We note that the model does not resolve these quantities on a fine (μm) scale, but uses coarse-grained values. These coarse-grained values can be interpreted as meso-scale averages over a number of pores in the scaffold material. For a scaffold that is additively manufactured based on a unit-cell design, the scaffold volume fraction is a spatially varying parameter that changes on a larger length-scale than the unit cell size. Similarly, the regenerated bone density and other quantities in our model can be seen as locally averaged quantities. See Fig. 1 for an illustration of a gyroid-type unit cell design. Other unit cell designs, for example, derived from shape optimization procedures, could of course be used. The basic underlying assumption of such homogenized models is that one can describe the evolution of these quantities again in terms of their averages.

The spatial domain of computation is the domain occupied by the scaffold. We simplify this domain to a one-dimensional object by only considering one of the main stress axes under physiological mechanical loading. This major reduction in complexity allows us to treat the resulting system of equations very efficiently, which is necessary for use in optimization. Of course, such a simplification requires some symmetry in the underlying three-dimensional setting such that the evolution of relevant quantities (e.g., regenerated bone density) can be captured in terms of quantities averaged over two of the three physical dimensions. The main drawback is that no optimization for the scaffold volume fraction can be performed in the two unresolved dimensions. Furthermore, only settings where there is one dominantly mechanically relevant axis can be treated. This is the case in our application, i.e., an example of a segmental defect in a long bone. See the section “Comparison to experimental results” for a comparison to experimental results for scaffold-mediated bone regeneration in an ovine model.

Concretely, we solve the following system of differential equations (in space on the domain (0, *L*) and time for *t* ∈ (0, *T*]) for (1) the scaffold volume fraction *ρ* = 1 − *θ* with *θ* being the scaffold porosity, (2) the mechanical displacement *u*, (3) the relative molecular weight of the scaffold material *σ* (normalized to be equal to unity for a new scaffold), (4) the density of bioactive molecules (growth factors/cytokines) *a* (normalized to unity for the density in healthy bone tissue under physiological mechanical loading), and (5) the relative bone density *b* (normalized to unity for healthy bone tissue).

1 $$\begin{array}{ll}{\rho }_{t}=0 & ({\rm{occupied}}\,{\rm{space}}\,{\rm{does}}\,{\rm{not}}\,{\rm{change}}\,{\rm{in}}\,{\rm{time}})\end{array}$$

2 $$\begin{array}{ll}0={({\mathbb{C}}(\rho ,\sigma ,b){u}_{x})}_{x} & ({\rm{mechanical}}\,{\rm{equilibrium}})\end{array}$$

3 $$\begin{array}{ll}{\sigma }_{t}=-\,{k}_{1}\sigma  & ({\rm{exponential}}\,{\rm{loss}}\,{\rm{of}}\,{\rm{molecular}}\,{\rm{weight}})\end{array}$$

4 $${a}_{t}={(D(\rho ){a}_{x})}_{x}+{k}_{2}|{u}_{x}|b-{k}_{3}a\,({\rm{diffusion}},\,{\rm{generation}},\,{\rm{and}}\,{\rm{decay}}\,{\rm{of}}\,{\rm{bioactive}}\,{\rm{molecules}})$$

5 $$\begin{array}{ll}{b}_{t}={k}_{4}a|{u}_{x}|(1-\frac{b}{1-\rho }) & \begin{array}{c}({\rm{bone}}\,{\rm{growth}}\,{\rm{proportional}}\,{\rm{to}}\,a\,{\rm{and}}\,{\rm{mechanical}}\,{\rm{strain}}\\ {\rm{but}}\,{\rm{constrained}}\,{\rm{by}}\,{\rm{free}}\,{\rm{volume}})\end{array}\end{array}$$

In this system, *k*_1_, *k*_2_, *k*_3_ and *k*_4_ are constant parameters, *D*, and ${\mathbb{C}}$ are functional relationships, all to be determined by experiment. Here, and in the remainder of the article, the subscript *t* denotes a time derivative, the subscript *x* denotes a spatial derivative.

The initial and boundary conditions for the respective variables are given by

6 $$\begin{array}{ll}\rho (x,0)={\rho }_{0}(x) & {\rm{for}}\,{\rm{all}}\,x\in ({\rm{0}},L)\end{array}$$

7 $$\begin{array}{ll}\sigma (x,0)=1 & {\rm{for}}\,{\rm{all}}\,x\in (0,L)\end{array}$$

8 $$\begin{array}{ll}a(x,\mathrm{0)}=0 & {\rm{for}}\,{\rm{all}}\,x\in ({\rm{0}},L)\end{array}$$

9 $$\begin{array}{ll}a\mathrm{(0,}\,t)=a(L,t\mathrm{)=1} & {\rm{for}}\,{\rm{all}}\,t\in [0,T]\end{array}$$

10 $$\begin{array}{ll}u\mathrm{(0},t)=0,u(L,t)=\gamma L & {\rm{for}}\,{\rm{all}}\,t\in [{\rm{0}},T]\end{array}$$

11 $$\begin{array}{ll}b(x,\,\mathrm{0)}=0 & {\rm{for}}\,{\rm{all}}\,x\in (0,L),\end{array}$$

i.e., in the beginning, the scaffold material with its given density (6) is fully intact (7), and no regenerated bone or active molecule is present (11), (8). The bone adjacent to the scaffold, however, produces the active molecules such that saturation is achieved there (9). The value for *u*(*L*, *t*) was chosen such that the scaffold is subject to a hard-loaded engineering strain *γ* (10). We note that the magnitude of the displacement boundary condition is arbitrary and could be normalized to unity by changing the parameter *k*_4_.

We note that our model includes local production of bioactive molecules only. An addition of a bone growth booster in the model could be implemented by changing the initial conditions for the function *a*. However, minimizing the required booster is relevant in particular for patients suffering from bone cancer.

### Model parameters

This model assumes high molecular weight (M_w_) PCL (~80 kDa) as the scaffold material. *In vivo*, PCL shows a two-stage degradation pattern, predominantly by bulk erosion. The first stage involves a decrease in M_w_ without volume loss. The second stage of degradation begins when the M_w_ drops below 8000 Da, at which point the material becomes brittle and loss of volume occurs^26^, but this happens beyond the time scale considered here. Therefore, we consider no loss of volume fraction of the scaffold material to occur over time, yielding eq. (1) with no additional parameters.

The other parameters used for the model are listed in Table 1. They were determined as follows.

- In humans, segmental bone defects are considered critical-sized when >2.5 cm^27^. In our model, the length of the scaffold was set at 30 mm, recapitulating a realistic critical-sized bone defect, as for example studied in an ovine model in^28^.
- In a healthy individual, it is expected that with the appropriate clinical interventions, bone defects should be completely bridged with low to medium weight-bearing capacity after 6 months^29^. The process of bone remodeling would follow and may take 3 to 5 years to complete restoration of the full function of the bone^30^. Our current model considers bone regeneration over a span of 12 months, capturing the critical phase for scaffold-mediated healing with the scaffold acting as the support across the defect site in the initial phase until the formation of clinically functional bridging across the bone defect.
- The PCL absorption rate constant, *k*_1_, was derived from experimental findings of Pitt *et al*.^31^, where PCL implanted into rabbits showed that after one year approximately 30% of the original M_w_ remains.
- The constants *k*_2_ and *k*_3_, which govern the generation and decay rate of the bioactive molecules, are more difficult to obtain from the literature. The systemic half-life of recombinant human-bone morphogenetic protein-2 (rhBMP-2) was reported to be 16 minutes in rats and 6.7 minutes in non-human primates after IV administration, while BMP-7^32^ and vascular endothelial growth factor (VEGF) exhibit a systemic half-life of 30 minutes in rats^33^. Nonetheless, it has been widely shown that the local retention of bioactive molecules is prolonged in the presence of scaffolds^34^. For example, Kim *et al*.^35^ showed that PCL/PLGA scaffolds exhibit a sustained release of BMP-2 for 28 days *in vitro* – but such data is not necessarily applicable to our setting as we do not consider scaffolds functionalized with bioactive molecules for controlled release. Presently, there is a lack of experimental data on the generation and decay rate of bioactive molecules in the present of PCL scaffolds in the defect site. Our value for *k*_2_ corresponds to a half-life of approximately 6 hours, which yields a good agreement when comparing the model’s resulting regenerated bone density to experimental outcome, see “Comparison to experimental results”. For comparison, the simulation results for a half-life of approximately 30 minutes are discussed in the appendix “Further simulation results”, item *(a)*, in the Supplementary Materials. The parameter *k*_3_ is set such that, for a constant bone density of 1 (i.e., healthy bone tissue) the equilibrium state for *a* is 1 as well (i.e., the normalized active molecule density in healthy bone tissue).
- The diffusivity constant, *k*_5_ in *D*, is a standard value for the diffusion of bioactive molecules^36^ that is typically measured for soluble proteins^37^. In the functional relationship *D*(*ρ*) = 1 − *ρ* we account for the increased tortuosity of the microscopic diffusion domain due to the scaffold.
- The relative modulus of bone vs. scaffold, *k*_6_, is chosen to agree with measurements (i.e, the ratio of the nano-indentation hardness for regenerated bone [^28^, Fig. 8(C), scaffold only] of 0.64 GPa and the nano-indentation hardness for PCL [^38^, Table 5]) of 0.071 GPa. For the elastic modulus ${\mathbb{C}}$ we use the Voigt bound for composites, which simply corresponds to the sum of the elastic moduli of the scaffold and the regenerated bone, weighted by their respective volume fraction.
- Finally, the regeneration parameter *k*_4_ is set such that a realistic regeneration outcome is achieved, see the section “Comparison to experimental results”.

**Table 1:** Parameters used for the bone regeneration model.

Param.	Value	Description
*T*	12 months	Period of bone regeneration considered in the model
*L*	30 mm	Length of bone defect/scaffold considered in the model
*γ*	1%	Hard-loaded engineering strain
*D*(*ρ*)	*k*_5_(1 − *ρ*)	Diffusivity of bioactive molecules in the scaffold occupied defect site
${\mathbb{C}}(\rho ,\sigma ,b)$	*ρσ* + *k*_6_*b*	Elastic modulus for the scaffold/regenerated bone material composite
*k*_1_	0.1 per month	PCL absorption rate constant
*k*_2_	*k*_3_/*γ*	Generation rate of bioactive molecules
*k*_3_	80 per month	Decay rate of bioactive molecules
*k*_4_	0.25/*γ* per month	Rate of bone regeneration (healthy individual)
*k*_5_	100(μm)^2^/s ≈ 260 (mm)^2^/month	Diffusivity of bioactive molecules without scaffold
*k*_6_	9.0	Relative modulus of bone vs. scaffold

### Numerical implementation

In order to obtain a numerical approximation to the solution of our model, we employ a simple first-order-in-time Euler scheme, where in each time step the discrete equations are solved in the order that they are displayed above (with current values for the quantities substituted in each equation), and are thus decoupled. The diffusion equation for *a* as well as the elliptic equation for *u* are discretized in space using piecewise affine finite (i.e., P1) elements in one space dimension, see^39^ or^40^. The resulting ordinary differential equations for *σ* and *a* are discretized implicitly in time, the equation for *b* is discretized explicitly in time. The time-step is 0.1 months and the spatial discretization length is 0.4 mm. For details regarding the numerical implementation we refer to the MATLAB code, which is available as Supplementary Material.

### Comparison to experimental results

**Figure 2:**
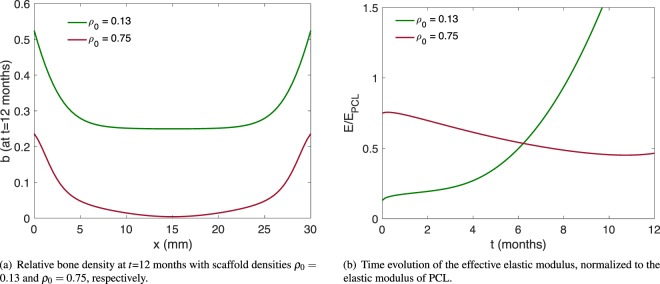
Outcome of the model without optimization.

The resulting regenerated bone matrix for the given parameters and two different constant values for the scaffolds’ volume fraction (*ρ*_0_ = 0.13 and *ρ*_0_ = 0.75, respectively) are shown in Fig. 2(a). Using the parameters from Table 1, and a constant scaffold volume fraction of 13%, our simulation yields a regenerated bone density of approximately 25% in the central region of the defect site (Fig. 2(a), green line). We note that an overly high volume fraction of scaffold material inhibits bone regeneration in our model (Fig. 2(a), red line), which is indeed an observed phenomenon in many studies (reviewed in^41^).

**Figure 3:**
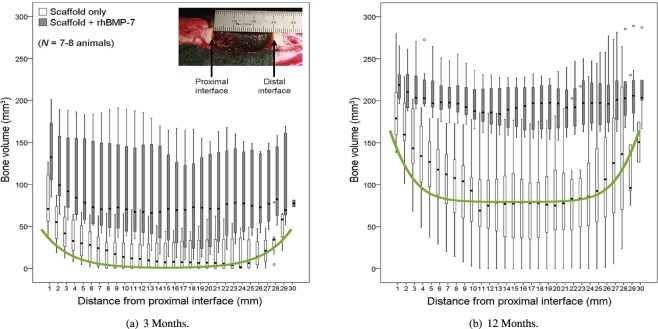
Experimentally determined regenerated bone volume, from^28^, reproduced and adapted with permission. Since we consider bone regeneration without the aid of rhBMP-7, we are interested in the scaffold-only data (light colored bars). The box plots indicate median, 25th and 75th percentiles, respectively. The vertical lines indicate maximum and minimum values. The green line represents the respective simulation results (as displayed in Figs 2(a) and 4(a)), scaled appropriately.

**Figure 4:**
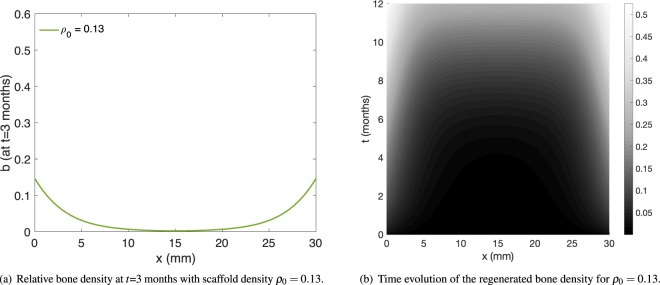
Regenerated bone density and its space and time dependence.

Detailed quantitative data for bone regeneration in the presence of a scaffold is unfortunately scarce. Our main comparison of the model outcome with experiment is thus limited to^28^. As can be deduced from [^28^, Fig. 1A,B], the scaffold used in the study done in^28^ has a volume fraction of approximately 13%. In Fig. 3 (reproduced from^28^ with permission), the experimentally determined regenerated bone volume is shown after 3 and 12 months of scaffold-mediated bone regeneration in an ovine model. The relevant “scaffold only” regeneration data after 12 months (Fig. 3(b)) shows a bone volume of approximately 75 mm^3^ in the central region. For a sample diameter of 20 mm and slice height of 1 mm, as given in^28^, this corresponds to an averaged density of $\frac{75\,{{\rm{mm}}}^{3}}{{(\frac{20}{2}{\rm{mm}})}^{2}\pi \cdot 1\,{\rm{mm}}}\approx 25 \%$, the same value our model yields. We also note that the overall shape – with a rather flat region of a constant amount of regenerated bone matrix in the central region – compares well to our results in Fig. 2(a), green line (*ρ*_0_ = 0.13). More quantitatively, we note that out of the 30 data points displayed in Fig. 3(b), for all but six of them, our simulation result lies within the first and third quartile box plots (after converting to regenerated densities as above). For all data points, our simulation result lies between the maximal and minimal experimental values. We note that the matching to the experimental data is slightly better at the distal end (*x*-values above 25 mm) than at the proximal end (*x*-values below 5 mm). It may therefore be possible to improve the matching by introducing spatially dependent model parameters – this, however, is beyond the scope of this article, as our focus lies on presenting an optimization method. Furthermore, one can compare the three month experimental data presented in Figs 3(a) to 4(a), which shows the regenerated bone density in our model after three months. The regenerated bone density in the central region of the defect is nearly zero in both experiment and simulation. Again, the model results lie within the first and third quartile for all but six experimental data points. The entire time and space dependence of the regenerated bone density is shown in Fig. 4(b). For comparison, the simulation results are overlaid in Fig. 3 for both the 3 month and the 12 month data. We note that the regenerated bone density increases with time extending from the proximal and distal ends of the defect parallel to the long bone axis. This pattern of scaffold-mediated bone regeneration has been reported previously^21,28^.

## Scaffold Density Optimization

Using current additive manufacturing technologies, it is possible to generate unit-cell based scaffolds with any given porosity distribution *ρ*_0_ on the domain occupied by the implant. We note that this distribution of porosity is a major aspect of the scaffold architecture. The solution of the evolution equations in our model now allows the deduction of, for example, the rate of proliferation of newly generated bone matrix within the implant, all dependent on the initial scaffold porosity distribution.

The model above can furthermore be used to evaluate the time evolution of the mechanical stiffness (i.e., effective elastic modulus of the structure stemming from both the scaffold material and the regenerated bone) of the implant for a given *ρ*_0_. This mechanical stiffness is (in our case) proportional to the total elastic energy in the system at time *t* ∈ [0, *T*],

12 $${E}^{{\rm{el}}}(t)={\int }_{0}^{L}\,{u}_{x}(x,t){\mathbb{C}}(\rho (x,t),\sigma (x,t),b(x,t)){u}_{x}(x,t)\,{\rm{d}}x,$$

where *ρ*, *σ*, *u*, and *b* follow Eqs (1–5) above (and thus depend on *ρ*_0_). We note that the elastic energy in the system is given by the integral over the elastic energy density, which in turn is given by the strain *u*_*x*_ (the spatial derivative of the displacement *u*) multiplied by the stress, i.e., the elastic modulus ${\mathbb{C}}$ multiplied again by the strain. Figure 2(b) shows the evolution of *E* in time for the two unoptimized test cases.

It is now possible to maximize the minimum over the regeneration time of the overall mechanical stiffness

13 $${E}^{{\rm{\min }}}({\rho }_{0})=\mathop{{\rm{\min }}}\limits_{t\in [0,T]}{E}^{{\rm{el}}}(t)$$

of the scaffold among all physiologically suitable (e.g., with a cutoff to ensure *ρ*_0_(*x*) ≤ 0.7 in order to not prevent vascularization) initial scaffold porosity distributions.

It is important to note that for PCL scaffolds such an optimization is a reasonable approach. For certain titanium-mesh scaffolds it has been found that softer scaffolds yield better regeneration results^24^ – PCL, however, is already a material that is less stiff than bone. Therefore, it is not an issue here to obtain sufficient mechanical stimulus for bone regeneration. Instead, the main drawback of PCL scaffolds is their potential for mechanical failure, which can be minimized by maximizing their mechanical stiffness.

We implement this optimization by the established method of using a gradient flow with respect to the objective function *E*^min^. To be more precise, we calculate an approximation of the Fréchet derivative ${\delta }_{{\rho }_{0}}{E}^{{\rm{\min }}}({\rho }_{0})$ (in the discretized setting, this is the gradient of *E*^min^ with respect to the coefficients in the spatial discretization of *ρ*_0_) by using finite differences in each node of the discretization of *ρ*_0_, i.e., one coefficient in the discretization of *ρ*_0_ is varied slightly and the resulting difference in *E*^min^ is stored as the finite difference with respect to this coefficient. Then a gradient ascent with a soft pointwise constraint on the volume fraction is performed, where the soft constraint is implemented by adding a penalty function to the objective, which attains large negative values once *ρ*_0_ becomes too large. The method is illustrated in the flowchart in the appendix “Flowchart of the optimization algorithm” in the Supplementary Materials. The computational cost for such an optimization method is easily manageable due to the use of coarse grained values in the evolution equation system. Our MATLAB implementation performs a full optimization and reaches a stationary state of the gradient ascent in a few minutes on a regular desktop computer, thus more sophisticated methods were not deemed necessary. Our method can therefore yield an optimized scaffold design, adapted to a given mechanical load and other (possibly patient specific) parameters.

## Results and Discussion

We have devised a simple model for bone regeneration that is suitable for use in an optimization routine for polymer bone scaffolds. Generally, one can establish a hierarchy of models for bone regeneration, from fully resolved microscopic models treating the entire device, to homogenized (coarse grained) models in three, and finally in one space dimension. Fully resolved models for bone regeneration, however, tend to be too computationally expensive to be used for optimization, as the computation time for one single run of such a model can be on the order of 30 minutes^42^ on a large scale (56 thread) compute server, and more than 100 runs can be necessary to determine an optimal scaffold^43^. A different route of simplification is to restrict oneself to a single unit cell in a periodic scaffold design. This approach is taken for example in^13,44^. The advantage of our coarse grained method is that, while neglecting the microscopic properties, the entire device can be treated numerically and optimized within minutes on a standard desktop machine.

Generally, we remark that our model can be verified using experimental data by comparing the time dependent regenerated bone density, which can be assessed from tomography results in *in-vivo* studies. Such a comparison was performed in the section “Comparison to experimental results”.

In our model, as a proxy for the mechanical stability of such scaffolds, we maximize the effective elastic modulus of the combination of scaffold and regenerated bone material, after taking the minimum effective elastic modulus over the regeneration time. With this method, it is possible to find optimized volume fraction distributions for additively manufactured scaffolds based, e.g., on periodic unit cell designs (Fig. 1). These unit cell designs could, for example, stem from the aforementioned periodic topology optimization methods. In addition to the effective elastic modulus of the device, we implement a penalty for overly large scaffold volume fraction, as this would prevent for example vascularization (a rather shorter time-scale process which is not directly captured in our model). A lower bound for the scaffold volume fraction does not need to be implemented explicitly, as such a local weak point would be directly captured in a diminished effective elastic modulus.

**Figure 5:**
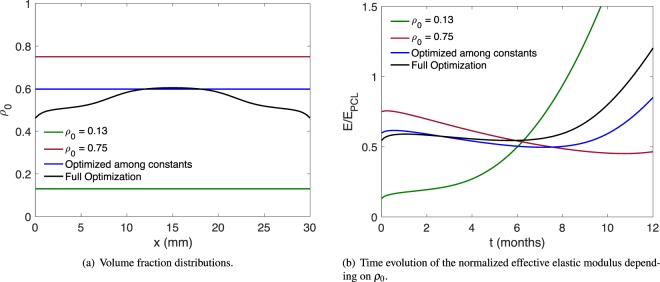
Comparison of scaffold optimization results.

Figure 5 shows a comparison of the optimized scaffold designs (Fig. 5(a)) and the corresponding normalized effective elastic moduli over time (Fig. 5(b)). The minimal elastic modulus values for all designs are listed in Table 2. In particular, we also compare the optimization among constant-volume fraction scaffolds. In our model, with the parameters given in Table 1, the difference in the minimal elastic modulus between the full optimization and the optimization restricted to constant volume fractions is approximately 8%. The full optimization yields an optimal scaffold with higher density in the middle region – this can, of course, be understood from heuristic arguments: the regenerated bone grows back at the ends where the scaffold is attached to the intact bone matrix. Thus, in the central region, the scaffold polymer has to maintain the structural integrity for a longer time by itself, while undergoing bulk erosion.

**Table 2:** Comparison of minimal elastic modulus *E*^min^(*ρ*_0_) for the scaffold designs under consideration.

*ρ*_0_ = 0.13	0.13
*ρ*_0_ = 0.75	0.45
Optimization among constant *ρ*_0_	0.50
Full optimization	0.54
Optimization among constant *ρ*_0_ (reduced bone regeneration)	0.26
Full optimization (reduced bone regeneration)	0.36
Reduced bone regeneration but *ρ*_0_ optimized for regular patient	0.28

The values are normalized such that the scaffold material PCL has an elastic modulus of 1.

We remark that our model is still very simplistic and a number of parameters are not known exactly due to the lack of quantitative experimental data. However, the aforementioned general outcome of optimal scaffold designs that increase their volume fraction in their central region (in order to provide more stability until the regenerated bone reaches the center) and decrease their volume fraction at the edges (in order to not impede bone-ingrowth) holds for a large range of different parameters. For illustration we discuss two more numerical examples for scaffold optimization in appendix “Further simulation results” in the Supplementary Materials.

**Figure 6:**
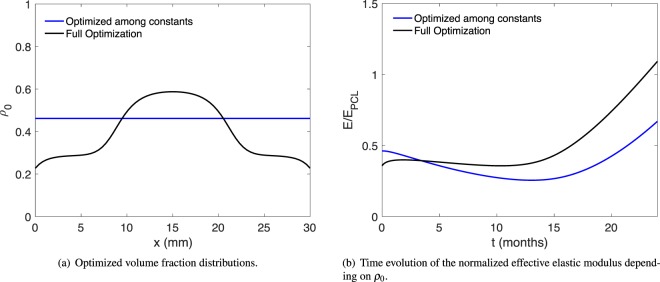
Comparison of scaffold optimization results for the model with reduced bone regeneration, i.e., *k*_4_ = 0.125/*γ* and *k*_6_ = 4.5.

In the real world, critical-sized bone defects are more prominent in patients with compromised endogenous bone healing capability, for example, due aging^45^ or bone-related metabolic disorders like osteoporosis^46^. Hence, we performed a test case for the generation of a scaffold with optimal porosity distribution for patients with compromised endogenous bone healing capability. This numerical experiment was conducted with the bone-regeneration coefficient reduced to *k*_4_ = 0.125/*γ* and the stiffness ratio to *k*_6_ = 4.5, to emulate a patient with reduced regeneration capacity, for example, due to osteoporosis. The minimal elastic modulus of the scaffold performed with full optimization and the optimization among constant *ρ*_0_ are listed in Table 2 – with a difference of nearly 40% (*E*^min^ = 0.36 vs. *E*^min^ = 0.26). The porosity distribution of scaffolds (for reduced bone regeneration capability) and the corresponding elastic moduli are plotted in Fig. 6.

We argued that the general shape of the optimal scaffold density distribution can be understood from heuristic arguments. It is thus particularly interesting to note the following. When we model the bone regeneration process for a scaffold optimized for a healthy patient (black line in Fig. 5(a), optimized for *k*_4_ = 0.25/*γ*, *k*_6_ = 9.0), but using model parameters of a patient with reduced regeneration capacity (*k*_4_ = 0.125/*γ*, *k*_6_ = 4.5), we get a significantly lower *E*^min^ = 0.28 than when we model a scaffold that has been optimized from the start specifically for a patient with reduced regeneration capacity (black line in Fig. 6(a), yielding *E*^min^ = 0.36 – an increase of nearly 29%). This shows that our model can benefit tremendously from patient-specific parameters to produce patient-specific optimal scaffold designs. A purely heuristic approach, based solely on the idea that a scaffold should be denser in the central region, would thus give an inferior result.

## Conclusions

We presented an optimization method for bone scaffold design, which is based on an efficient, coarse grained model for bone regeneration. The regeneration parameters in the model have been determined from the literature and the outcome of the model was compared to experimental results. The model presented here can of course be further extended to include spatially dependent regeneration parameters or more sophisticated models for the scaffold decay (for example by including a dependence on the strain), as more experimental data becomes available.

Presently, diagnosis of bone defects is highly dependent on X-ray micrographs, which only provide visual guidance for treatment and management and lack precise information on the patient’s intrinsic bone regeneration capability. Potentially, this could be overcome by leveraging Omics technology and multi-model analysis through bioinformatics techniques to improve patient stratification in terms of intrinsic bone regeneration capability while unraveling the underlying biological mechanisms that govern the bone regeneration cascade. The inclusion of such patient-specific parameters would significantly improve the herein proposed optimization model for personalized bone scaffold designs. Nonetheless, such an endeavor will require long-term synergistic and complementary research efforts across a multitude of disciplines.

Hence, within the scope of design optimization, future work includes refinement of the model in order to be better aligned with experimental results, considering also finely resolved descriptions of bone regeneration in the presence of scaffolds, e.g.^47^. An extension to a three dimensional coarse grained regeneration model is also currently being examined.

## Supplementary information

Appendix

Supplementary Dataset 1

## Data Availability

This work does not have any experimental data. The MATLAB-code for scaffold optimization is made available as Supplementary Material.
