# Supplementary material for: Optimization of Bone Scaffold Porosity Distributions
Source: Sci Rep. 2019 Jun 24;9:9170. doi: 10.1038/s41598-019-44872-2 (PMC6591284; doi:10.1038/s41598-019-44872-2)

# Optimization of Bone Scaffold Porosity Distributions (Appendices)

**Patrina S.P. Poh, Dvina Valainis, Kaushik Bhattacharya, Martijn van Griensven, and Patrick Dondl**

## Appendix: Further simulation results

We present two more settings for our model and optimization. These results can serve as guidance for future experimental studies in order to refine and calibrate the bone regeneration model.

*(a)* Figure A1 shows the results for the bone regeneration table with $k_3 = 960$ per month, which corresponds to a half-life of bioactive molecules of approximately 30 minutes. The parameter $k_4$ has been adapted such that the regenerated bone density after 12 months is still approximately 25% in the central region for the scaffold with 13% constant volume fraction, in order to match the experimental results in[28] (see Figure A1(a)). The other parameters are as given in Table 1 in the article. We note that the shallow region of low regenerated bone density extends further to the edges of the scaffold domain, which does not match the experimental results in Figure 3B in the article as well as the simulation displayed in Figure 2(a) (green line) in the article. The reported in-vivo systemic half-lives of approximately 30 minutes for some relevant bioactive molecules (see section "Model parameters", item *(d)*, in the article) are therefore not directly applicable in our model. This may be due to a stabilizing effect of the scaffold and bone matrix environment on the generated bioactive molecules.

Again, we do see an optimal scaffold with a denser central region. The overall effect of the scaffold optimization is somewhat more pronounced in the present regime, as one can see in Figure A1(b), where the fully optimized scaffold exhibits a greater density difference between the central and the edge regions compared to Figure 5(a) in the article. This is due to an even stronger localization of bone regeneration near the defect edges in the beginning. The improvement in the minimal elastic modulus is also larger in this setting (approximately 33% from 0.36 to 0.48 in the minimal elastic modulus of the scaffold optimized among constant volume fractions and the full optimization). The time evolution of the elastic moduli is displayed in Figure A1(c).

*(b)* Figure A2 shows the results for a larger defect site with a length of 40 mm. The other parameters are as given in Table 1 in the article. We note that, again, the overall shape of the fully optimized scaffold design exhibits a denser central region and thus conclude that this feature is indeed very stable under parameter changes. Naturally, the regenerated bone density in the central region is lower than 25% in the scaffold with constant 13% volume fraction (see Figure A2(a)) – but quantitative experimental data for such a defect is not available. The optimized scaffold designs and the time evolution of the elastic moduli are displayed in Figures A2(b), A2(c). We note a more pronounced optimization effect again in this scenario (approximately 27% from 0.37 to 0.47 in the minimal elastic modulus of the scaffold optimized among constant

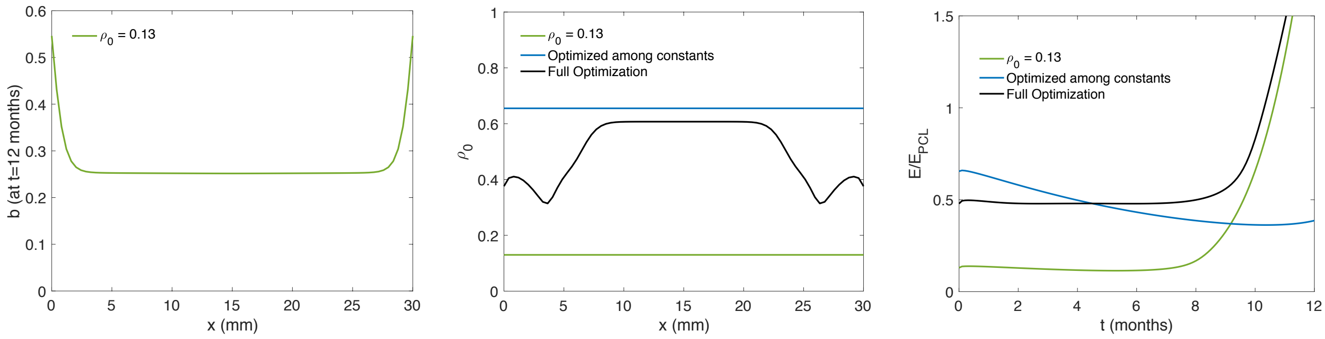


(a) Regenerated bone density for a standard scaffold with constant volume fraction.

(b) Optimized volume fraction.

(c) Elastic moduli time evolution.

**Figure A1.** Simulation and optimization results in a numerical model with reduced bioactive molecule half-life.

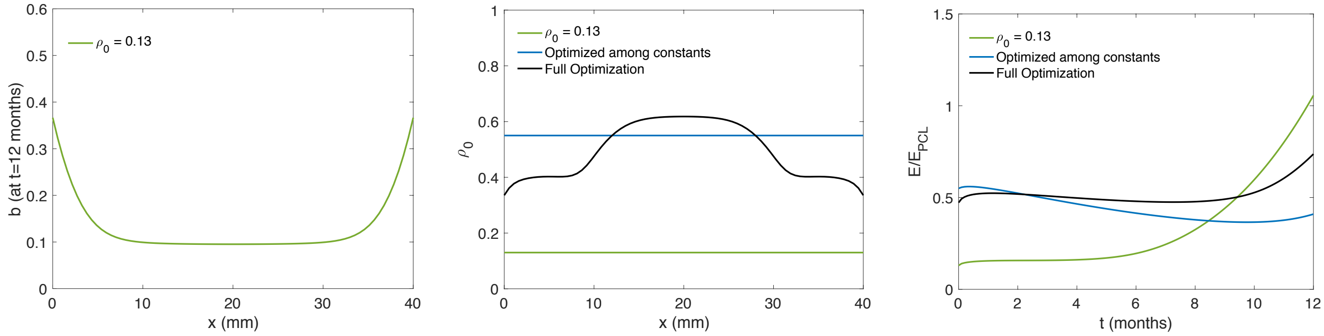


(a) Regenerated bone density for a standard scaffold with constant volume fraction.

(b) Optimized volume fraction.

(c) Elastic moduli time evolution.

**Figure A2.** Simulation and optimization results for a longer (40 mm) defect site.

volume fractions and the full optimization) and thus conclude that scaffold optimization may be become more relevant for more severe defects.

## Appendix: Flowchart of the optimization algorithm

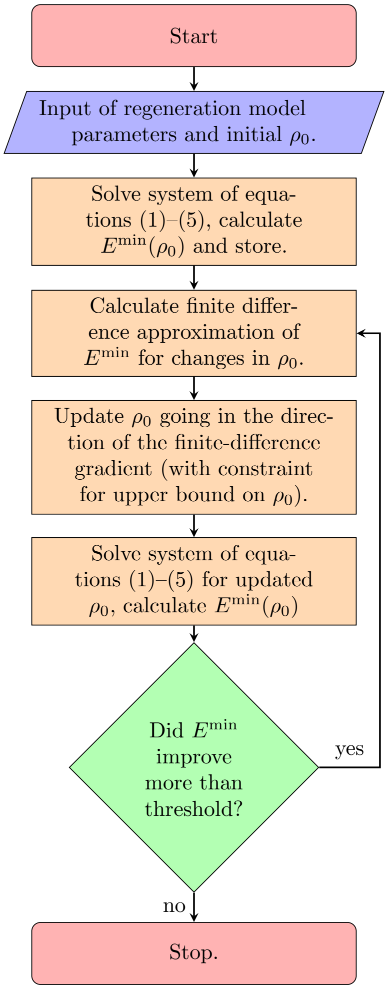

Supplement: Supplementary file 1 — Appendix [file 41598_2019_44872_MOESM1_ESM.pdf]
